# Supplementary material for: Loss of luminal lineage drives resistance to next-generation ERα antagonists in pretreated ER+ HER2− locally-advanced or metastatic breast cancer
Source: Nat Commun. 2026 Apr 1;17:4675. doi: 10.1038/s41467-026-71233-1 (PMC13201648; doi:10.1038/s41467-026-71233-1)
Supplement: Supplementary file 1 — Supporting Information [file 41467_2026_71233_MOESM1_ESM.docx]

**Supporting Information**

**Loss of luminal lineage drives resistance to next-generation ERα antagonists in pretreated ER+ HER2- locally-advanced or metastatic breast cancer**

Jackson Liang^1*^, Christy Ong^2^, Kareem Heslop^2^, Jane Guan^2^, Vasumathi Kameswaran^3^, Bence Daniel^3^, Minyi Shi^3^, Yuxin Liang^3^, Jennifer M. Giltnane^4^, Junko Aimi^1^, Ching-Wei Chang^5^, Mary R Gates^6^, Jennifer Eng-Wong^6^, Pablo Perez-Moreno^7^, Komal L. Jhaveri^8^, Nicholas C. Turner^9^, Elgene Lim^10^, Ciara Metcalfe^2^, Heather M. Moore^1^

# **Affiliations:**

# ^1^ Department of Translational Medicine, Genentech, South San Francisco, CA, USA.

# ^2^ Department of Discovery Oncology, Genentech, South San Francisco, CA, USA.

# ^3^ Department of Proteomic and Genomic Technologies, Genentech, South San Francisco, CA, USA.

# ^4^ Department of Research Pathology, Genentech, South San Francisco, CA, USA.

# ^5^ Department of Biostatistics, Genentech, South San Francisco, CA, USA.

# ^6^ Department of Early Clinical Development, Genentech, South San Francisco, CA, USA.

# ^7^ Department of Product Development, Genentech, South San Francisco, CA, USA.

# ^8^ Breast Medicine Service, Department of Medicine, Memorial Sloan Kettering Cancer Center, New York, New York, and Weill Cornell Medical College, New York, New York.

# ^9^ Royal Marsden Hospital and Institute of Cancer Research, London, UK.

# ^10^ St Vincents Hospital, University of New South Wales and Garvan Institute, Sydney, New South Wales, Australia.

*Corresponding author (liang.jackson@gene.com)

**Supplementary Figures**

#
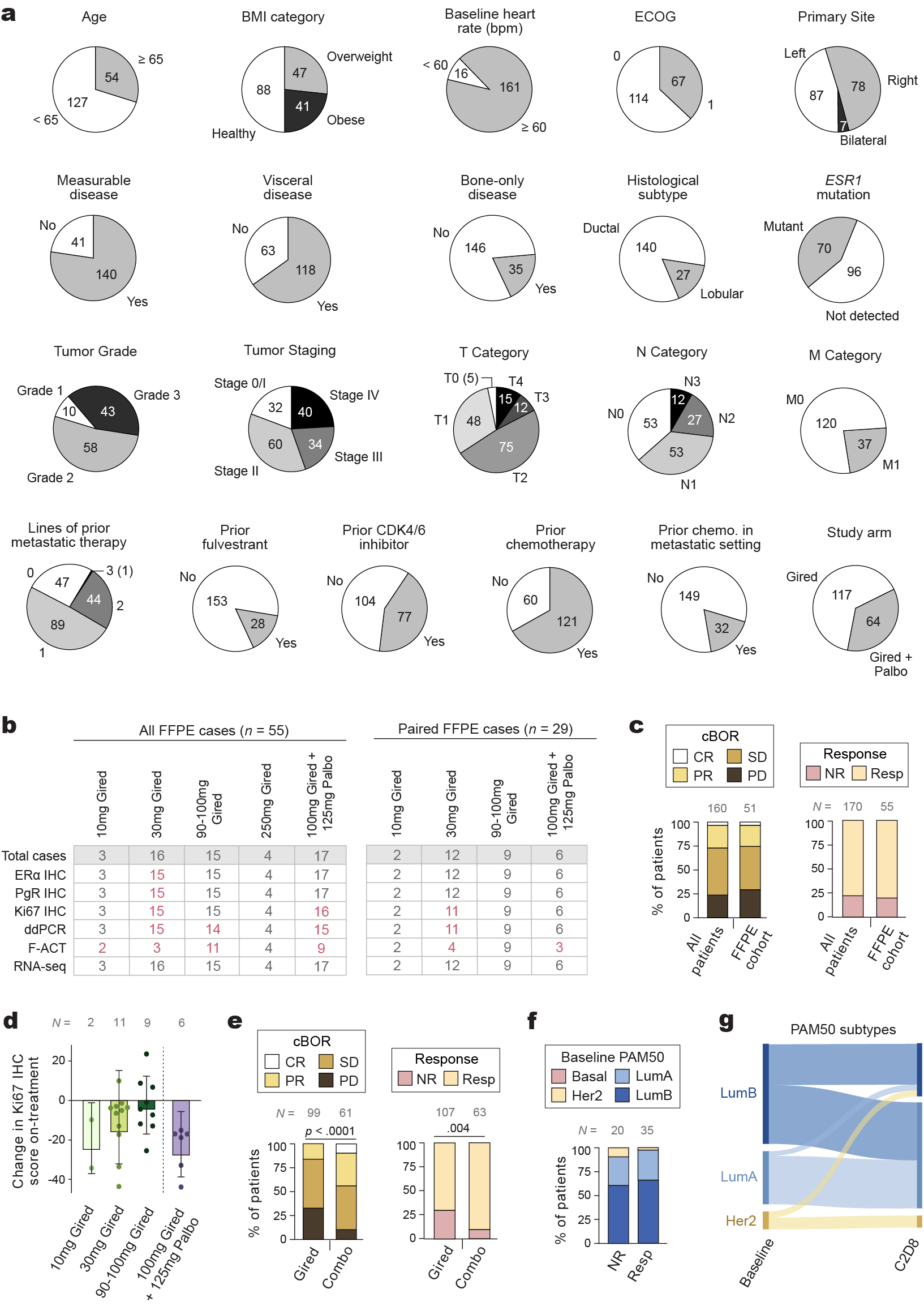


**Supplementary Figure 1. Cohort overview for study GO39932, a phase-1 study of giredestrant in pre-treated ER^+^ mBC patients.**

(**a**) Prevalence of key clinical features in study GO39932; a total of *n* = 181 patients were evaluated. Per clinical feature, pie charts show patients with a confirmed value; patients with missing data were excluded. For BMI, cutoffs of 25 and 30 were used to categorize patients as healthy, overweight, or obese. *ESR1* mutation status was evaluated centrally via digital droplet PCR (ddPCR). (**b**) Summary of available data from patients who provided FFPE specimens on this study. (**c**) Summary of confirmed best overall response (cBOR) via RECIST criteria and response (NR vs. Resp) for all evaluable patients or those with available FFPE specimens at baseline. (**d**) Change in KI67 score (%-positive cells) across treatment arms; each point is one patient. Negative percentages represent a decrease in KI67 upon treatment; values are geometric means ± geometric SD. (**e**) Summary of cBOR via RECIST criteria and response (NR vs. Resp) by treatment arm; *p*-values via two-sided χ^2^-test. (**f**) Prevalence of PAM50 subtypes at baseline by response. (**g**) Sankey plot of PAM50 subtypes per patient tumor at baseline vs. C2D8. Source data is available.

**
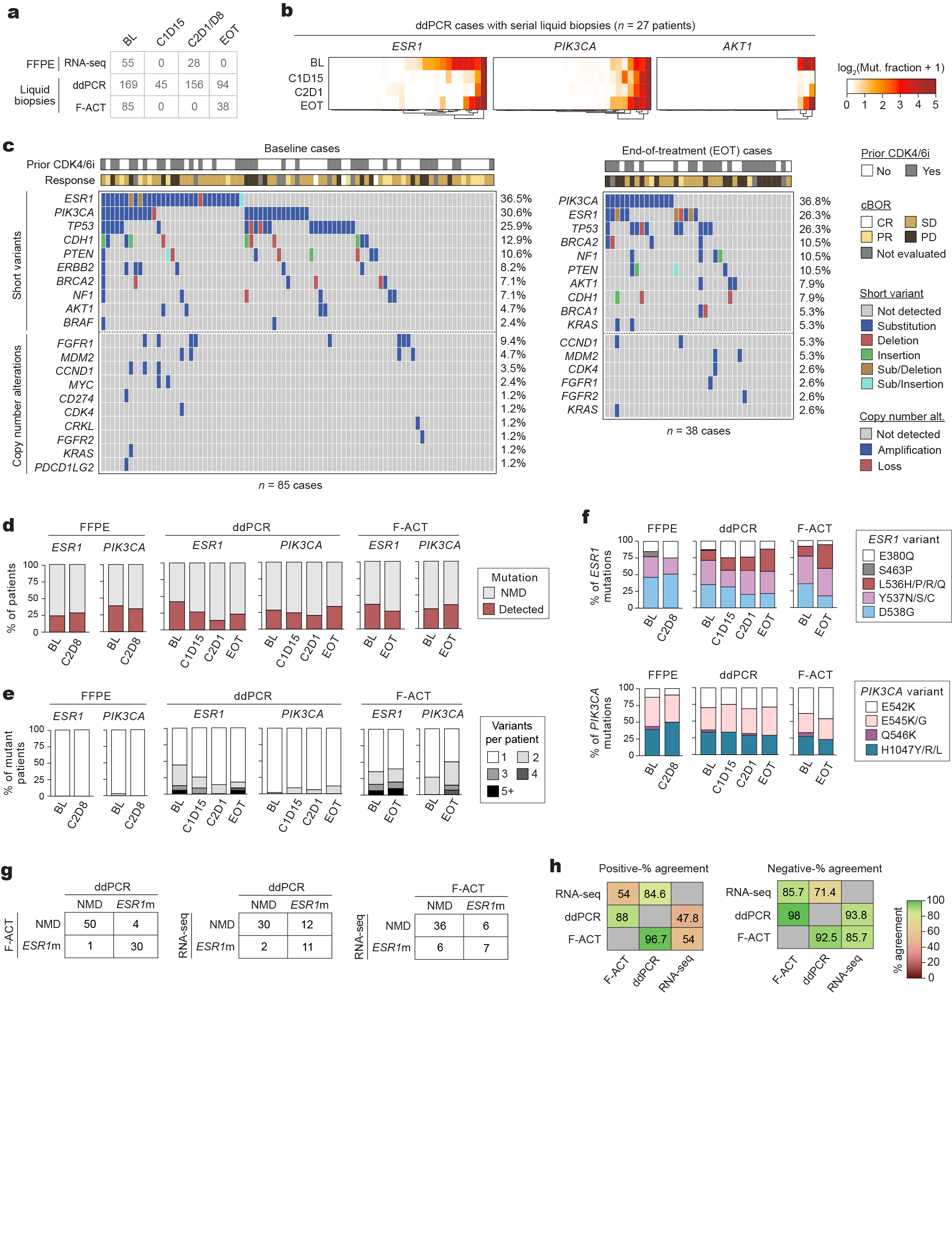
**

**Supplementary Figure 2. Concordance of mutational calls across assays.**

(**a**) Summary of mutational calls available per assay; ddPCR against *ESR1*, *PIK3CA*, and *AKT1*, FoundationACT (F-ACT) genomic profiling, and mutational inference via RNA-seq reads. (**b**) Heatmaps of ddPCR mutational fraction in cases for which data was available at all timepoints; each column is one patient. For patients carrying multiple variants in a single gene, the mutational fractions were summed. (**c**) Heatmaps of detected mutations via F-ACT panel, grouped by short variants or copy number alterations, and stratified by timepoint; each column is one patient. Confirmed best overall response (cBOR) by RECIST criteria. Percentages to the right of each row denotes the overall prevalence of each mutation. (**d**) The percentage of patients with mutations detected across assays. (**e**) For patients with a mutation detected, the number of unique variants across assays. (**f**) Prevalence of all detected variants per gene across assays. (**g**) Pairwise comparison of *ESR1-*NMD and *ESR1*m (mutant) calls per assay. For RNA-seq, samples are ‘mutant’ if the variant allele frequency represents at least 5% of total transcript. (**h**) Percent agreement for assay results in detecting *ESR1* mutations. Percent agreement is calculated for the assays in each row against those in respective columns. In (**c-h**), data are individual biopsies collected at specified visits and tested by respective assays; respective *n* are specified in panel (**a**). Source data is available.


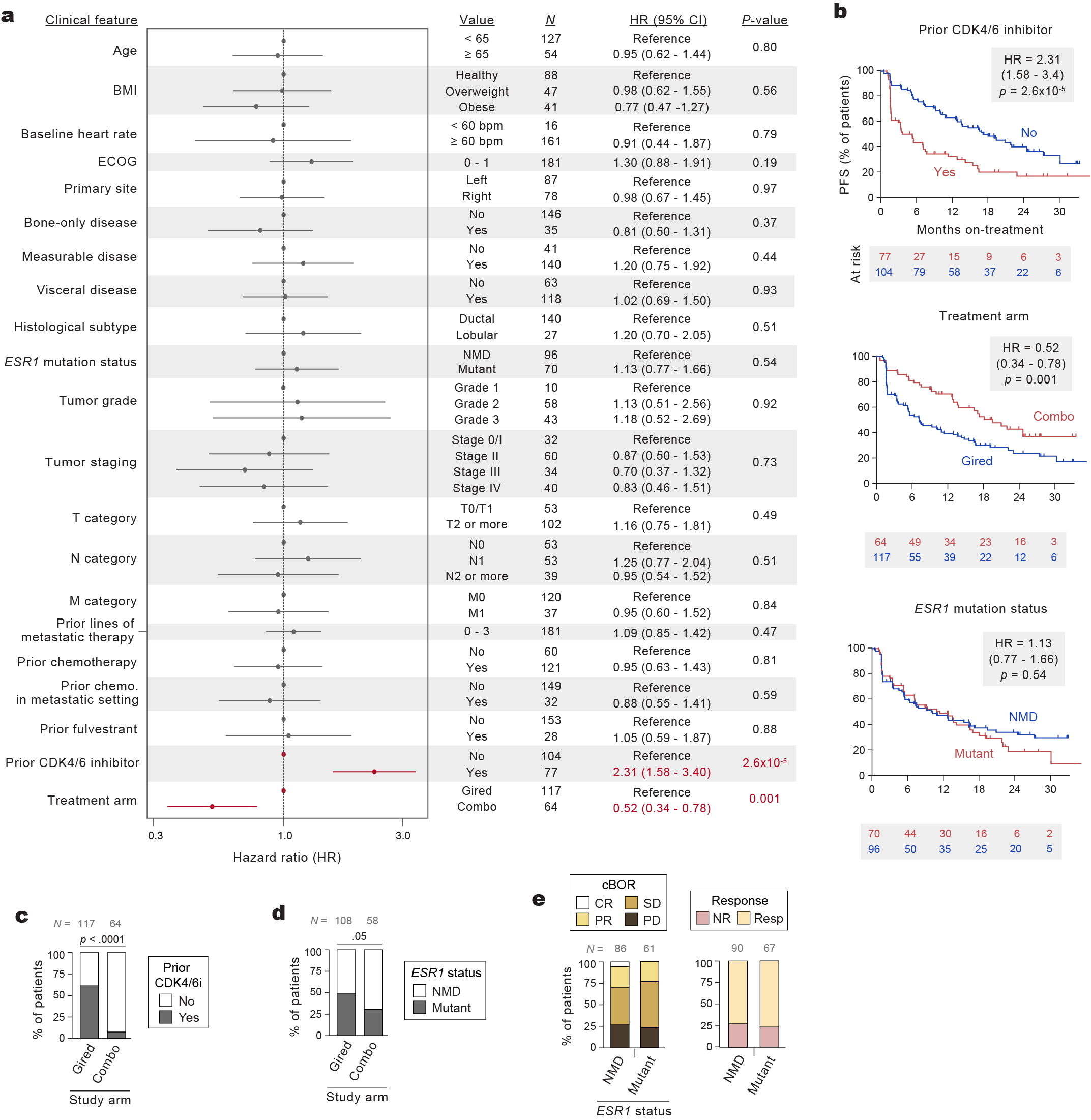


**Supplementary Figure 3. Clinical associations with progression-free survival.**

(**a**) Assessment of the association between key clinical features and progression-free survival (PFS) via Cox proportional-hazards model for *n* = 181 patients were assessed in total. Values are hazard ratios ± 95% confidence intervals (CI), computed using univariate Cox regression analysis. *P-*values were computed via likelihood-ratio test; significant variables are colored red. For categorical variables, reference is specified; for continuous variables, one HR was computed for the entire variable (no reference specified). (**b**) Comparison of PFS by select clinical variables; HR, 95% CI, and *p-*values were calculated in panel (**a**). For each plot, the variable colored in blue was used as the reference for regression analysis. (**c-d**) By study arm, frequency of treatment with prior CDK4/6 inhibitor (**c**) or *ESR1* status (**d**); *p*-values via two-sided χ^2^-test. (**e**) Summary of cBOR via RECIST criteria and response (NR vs. Resp) by *ESR1* status. In all panels, *ESR1* mutation status was evaluated centrally via digital droplet PCR (ddPCR); NMD: no mutation detected. For treatment arm, monotherapy giredestrant (Gired) and palbociclib combination (Combo). Source data is available.


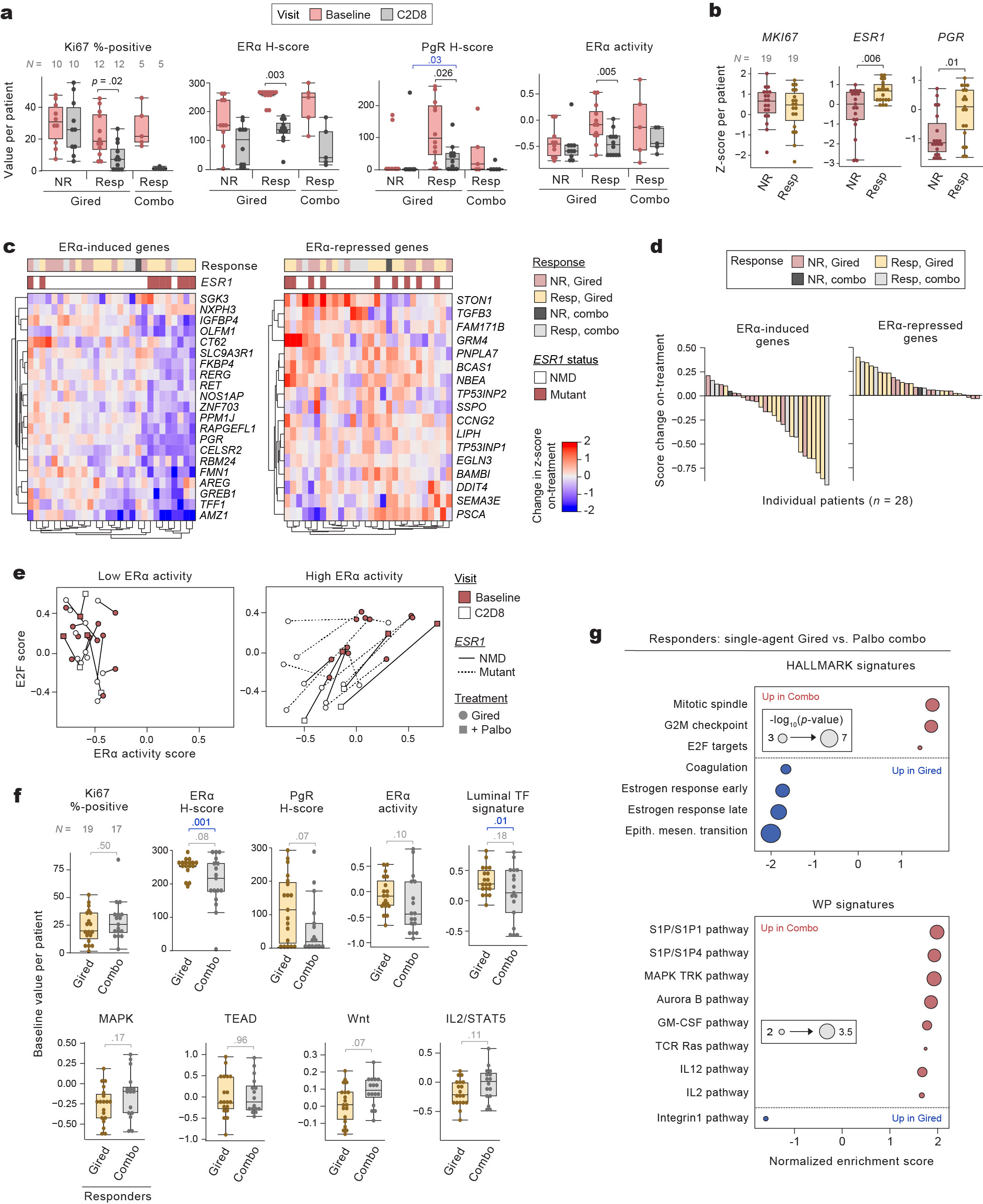


**Supplementary Figure 4. Analysis of baseline and on-treatment paired biopsies and comparison of responders across treatment arms.**

(**a**) Key biomarkers for paired baseline and on-treatment (C2D8) biopsies by response. *P*-values by two-sided Mann-Whitney *U-*test: black – paired tests per patient, blue – unpaired tests across response groups. One patient had NR in the palbociclib combination arm; patient was excluded from this analysis. (**b**) Expression of *MKI67, ESR1*, and *PGR* by response at baseline. *P*-values by unpaired two-sided Mann-Whitney *U-*test. (**c-e**) On-treatment changes in ERα activity half signatures; *n* = 28 patients. (**c**) Heatmaps of genes comprising half signatures; values are change in z-score per patient on-treatment. Each column is one patient and each row is one gene within the respective half-signatures. *ESR1* status was defined by RNA-seq calls on baseline tumors. (**d**) On-treatment change in ERα activity half-signature scores; each bar represents one patient. (**e**) Changes in ERα activity (using the ERα-induced signature) and E2F (cell proliferation) gene signatures on-treatment, stratified by baseline ERα activity. For baseline ERα activity groups, ‘high’ activity cases are defined as those equal or greater than the median ERα activity, and ‘low’ are those below the median value. Each point is one biopsy, and lines connect baseline and C2D8 biopsies per patient. *ESR1* mutational status was inferred by RNA-seq reads (mutant if the variant allele frequency is ≥ 5% of total transcript). (**f**) For responders, key biomarkers at baseline by treatment arm: monotherapy giredestrant (Gired) or giredestrant plus palbociclib combination (Combo). *P*-values: black/gray – unpaired two-sided Mann-Whitney *U-*test (grayed *p*-values are *p* > 0.05), blue – two-sided F-test. (**g**) Gene set enrichment analysis on HALLMARK and WikiPathways (WP) gene signatures for monotherapy vs. palbociclib combination baseline biopsies. Each point is one gene signature; *p*-values were computed using the ‘fgsea’ method. Pathways with FDR < 0.05 are shown. In (**a, b, f**), boxes are 25^th^ percentile, median, and 75^th^ percentile, whiskers are maximum/minimum values within 1.5-times the IQR. Each point is one baseline biopsy. Source data is available.


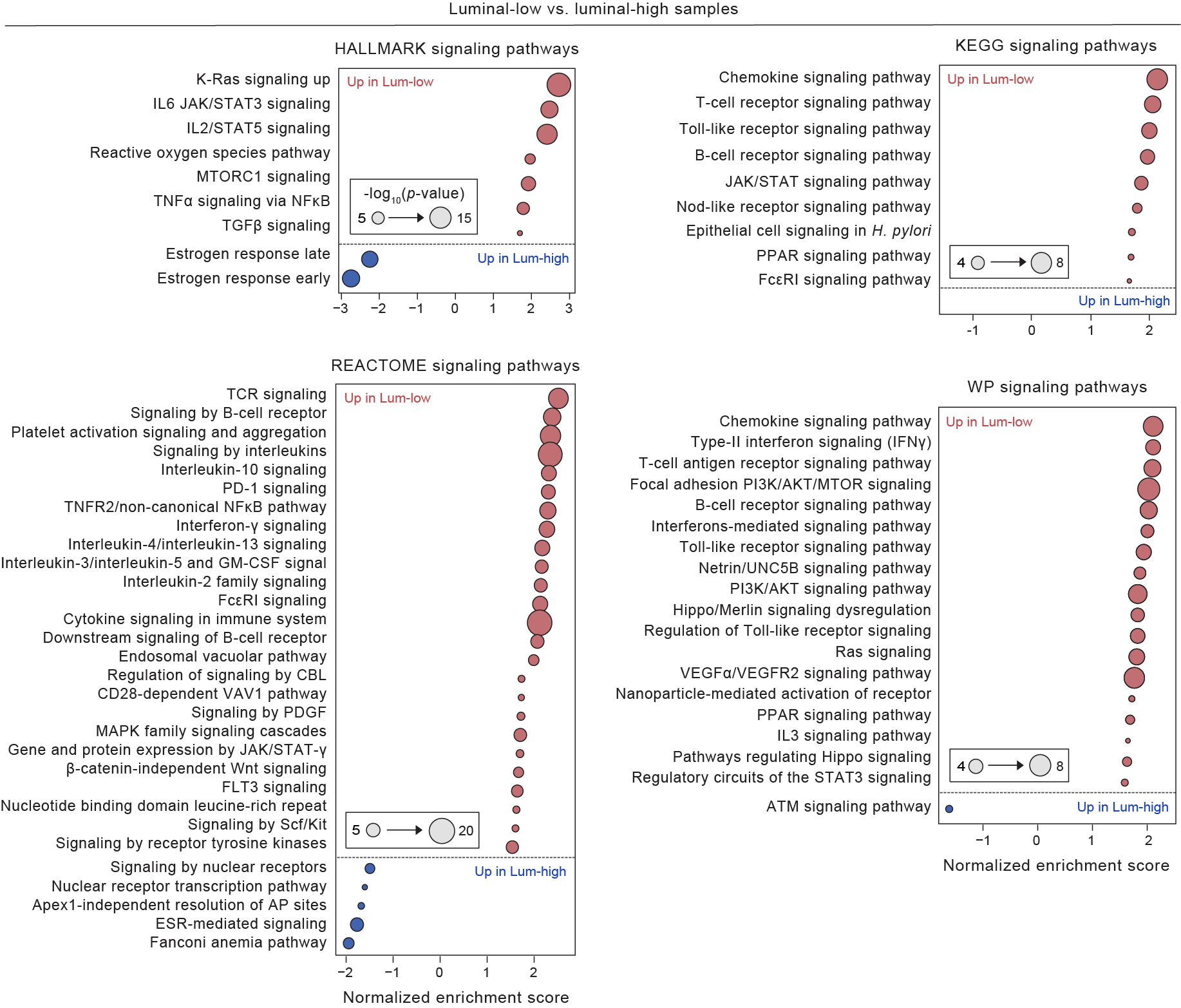


**Supplementary Figure 5. Enriched pathways in luminal-low vs. luminal-high biopsies at baseline.**

Analysis of gene signature enrichment for signaling pathways from specified collections, comparing baseline Lum-low and Lum-high cases (defined in Figure 1e). Pathways containing the terms ‘signaling’ and/or ‘pathway’ with FDR < 0.1 are shown. Each point represents one gene signature; *p-*values were computed using the ‘fgsea’ method. WP: WikiPathways. Source data is available.

**
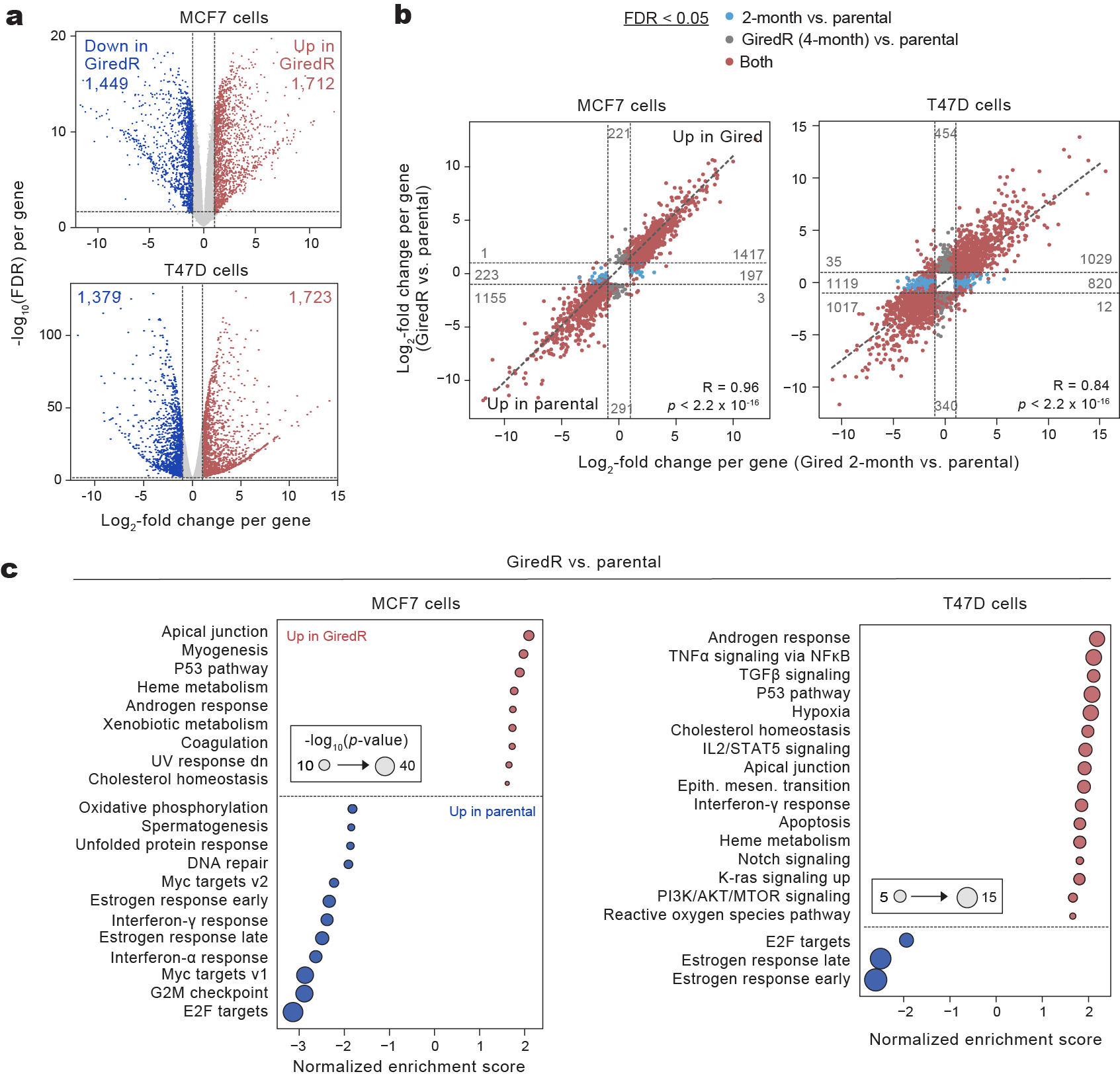
**

**Supplementary Figure 6. Additional RNA-seq analyses on parental and GiredR cell lines.**

(**a**) Volcano plots of differential gene expression between parental and GiredR cells by RNA-seq for MCF7 and T47D respectively. Each point is one gene. Colored points represent genes achieving a statistical cutoff of FDR < 0.05. Dotted lines: log_2_-fold change cutoffs of ±1, FDR cutoff of 0.05. (**b**) Comparison of differential gene expression between 2 month-treated cell lines vs. parental (x-axis) and GiredR (4 month-treated) cell lines vs. parental (y-axis). Data are represented in a 4-way plot, in which each point is one gene and each axis compares log_2_-fold change in expression between respective conditions and parental cells. Points are colored by whether genes satisfy an FDR cutoff of 0.05 in either condition compared to parental cells. Dotted lines denote log_2_-fold change cutoffs of ±1. (**c**) Gene set enrichment analysis of HALLMARK gene signatures between respective parental and GiredR cell lines. Each point is one gene signature; *p*-values were computed using the ‘fgsea’ method. Pathways with FDR < 0.05 are shown. In (**a-c**), *n* = 2 biological replicates per cell line. Source data is available.


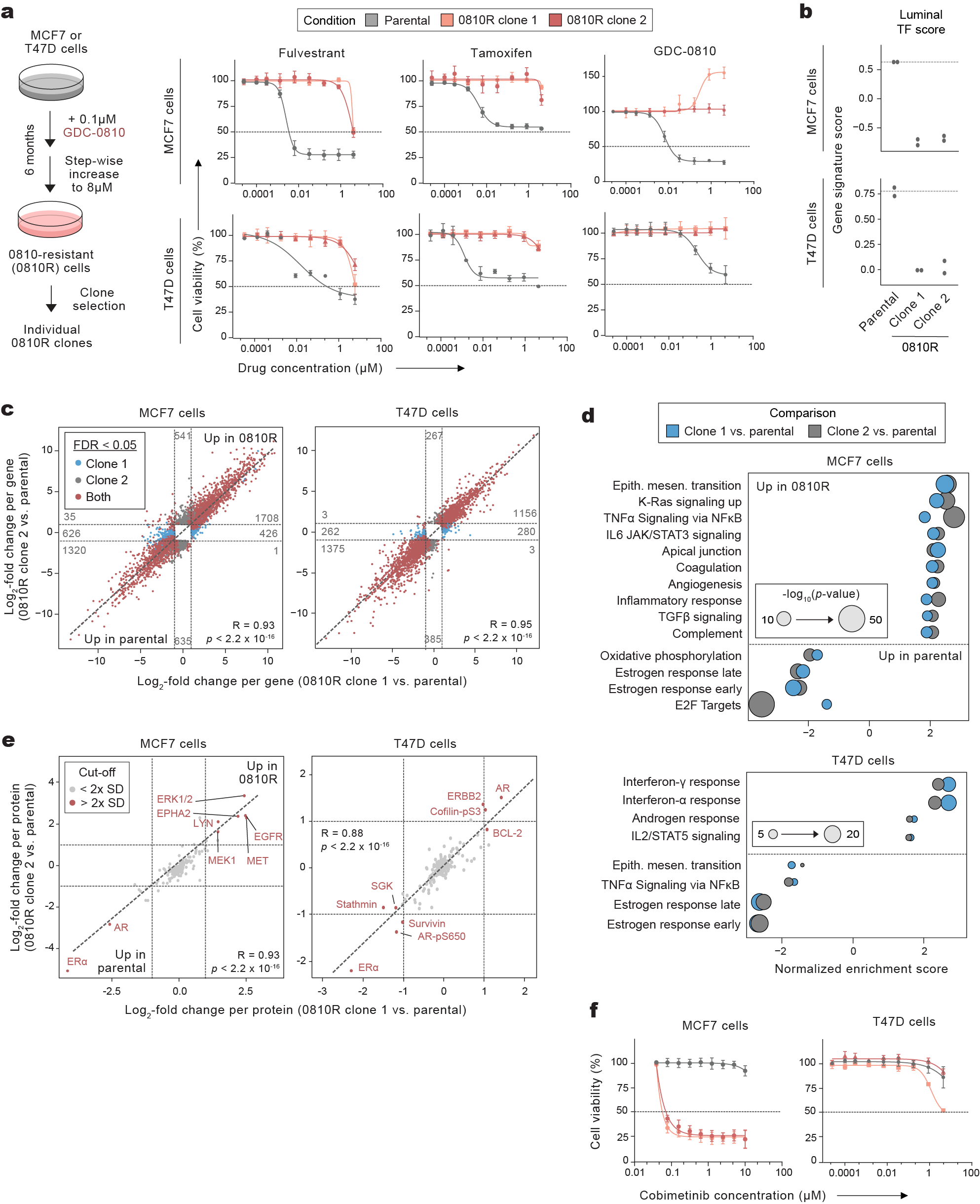


**Supplementary Figure 7. Analysis of GDC-0810-resistant (0810R) cell lines.**

(**a**) Generation of GDC-0810 resistant (0810R) MCF7 and T47D breast cancer cells. (Left) Experimental strategy; after resistant cells were established, two clones were selected per model for characterization. (Right) Cell viability for anti-ERα therapeutic ligands for parental and 0810R cells. (**b**) Luminal TF gene signature scores via RNA-seq per sample; each point is the calculated score for one sample. Dotted lines: mean value in respective parental cells. (**c**) Differential gene expression via RNA-seq between 0810R clones and parental cells per model. Each point is one gene. Each axis represents the log_2_-fold change in gene expression between an 0810R clone and parental cells. (**d**) Gene set enrichment analysis of HALLMARK gene signatures between respective parental and 0810R cells. Each point is one gene signature; *p*-values were computed using the ‘fgsea’ method. Pathways with FDR < 0.05 are shown. (**e**) Reverse phase protein array (RPPA) panel to detect differences in proteins and phospho-proteins between 0810R clones and parental cells per model; *n* = 1 biological replicate analyzed per condition. Each point is one protein species. Each axis represents the log_2_-fold change in protein abundance between an 0810R clone and parental cells. (**f**) Cell viability for cobimetinib in parental and 0810R cells. In (**a, f**), *n* = 3-4 biological replicates per cell line. Cell viability was measured using the CellTiterGlo assay; dotted lines: IC50 value (half-maximal inhibitory concentration) per drug. Values are mean ± error. In (**b-d**), *n* = 2 biological replicates analyzed per condition. In (**c, e**), dotted lines represent log_2_-fold change cutoffs of ±1. R and *p-*value via Pearson’s correlation method. Source data is available.


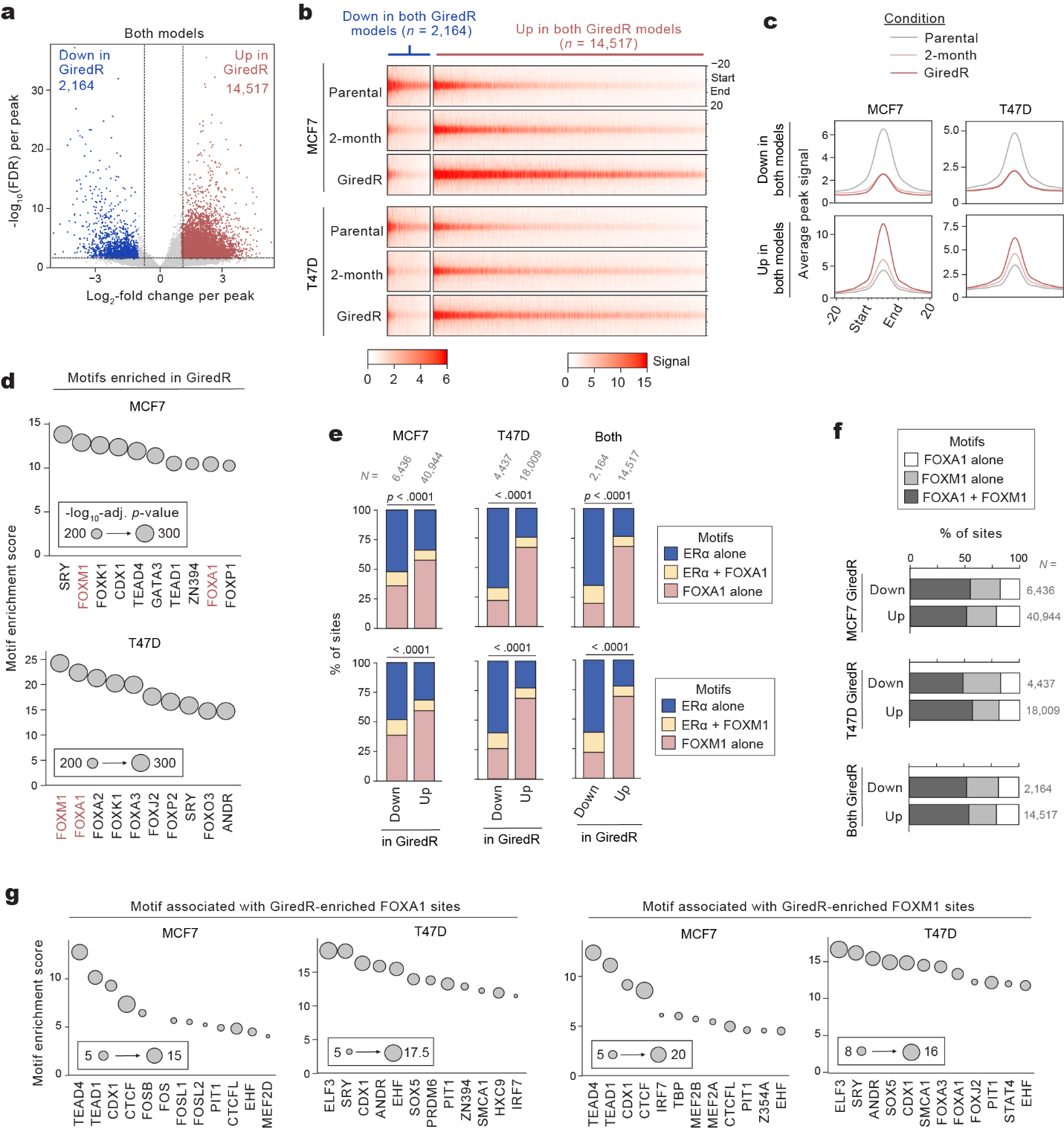


**Supplementary Figure 8. Additional ATAC-seq analyses on GiredR cell lines.**

(**a**) Volcano plot of differential ATAC peaks (chromatin accessibility) between parental and GiredR cells when pooling MCF7 and T47D models for analysis (*n* = 3 biological replicates per condition). Each point is one ATAC peak. Colored points represent peaks achieving a statistical cutoff of FDR < 0.05. Dotted lines: log_2_-fold change cutoffs of ±1, FDR cutoff of 0.05. (**b-c**) Differential ATAC peaks common between both MCF7 and T47D over treatment: parental, 2 months and GiredR (4 months). Data shows one sample per condition, which is representative of *n* = 3 biological replicates. (**b**) Heatmaps of differential sites per model. (**c**) Average ATAC peak signal per treatment. On the x-axis, peaks are normalized to ±20% of the total peak length. (**d**) Motif enrichment analysis for Gired-enriched motifs (up in GiredR; see Figure 4a-b) for each cell line; for merged analyses, see Figure 4d. Peaks which did not change between parental and GiredR (*n* = 118,999 and 126,294 respectively; defined by -1 < log_2_-fold change < 1 and FDR < 1) were used as the reference for enrichment analysis. (**e**) Representation of motifs for sites containing one or more of either ERα (ESR1) or FOXA1/FOXM1 motifs. (**f**) Representation of motifs for peaks containing one or more of FOXA1 or FOXM1 motifs. (**g**) Analysis of motif enrichment for the subset of ‘up in GiredR’ peaks which contain either a FOXA1 (top) or FOXM1 (bottom) motif for each cell line individual; for merged analyses, see Figure 4e. Per analysis, the corresponding set of FOXA1/FOXM1 motif-containing ‘down in GiredR’ peaks were used as the reference. In (**d, g**), each point is the enrichment score for one motif; *p*-values by one-sided Fisher’s exact test. In (**e, f**), *p*-values via two-sided χ^2^-test. Source data is available.


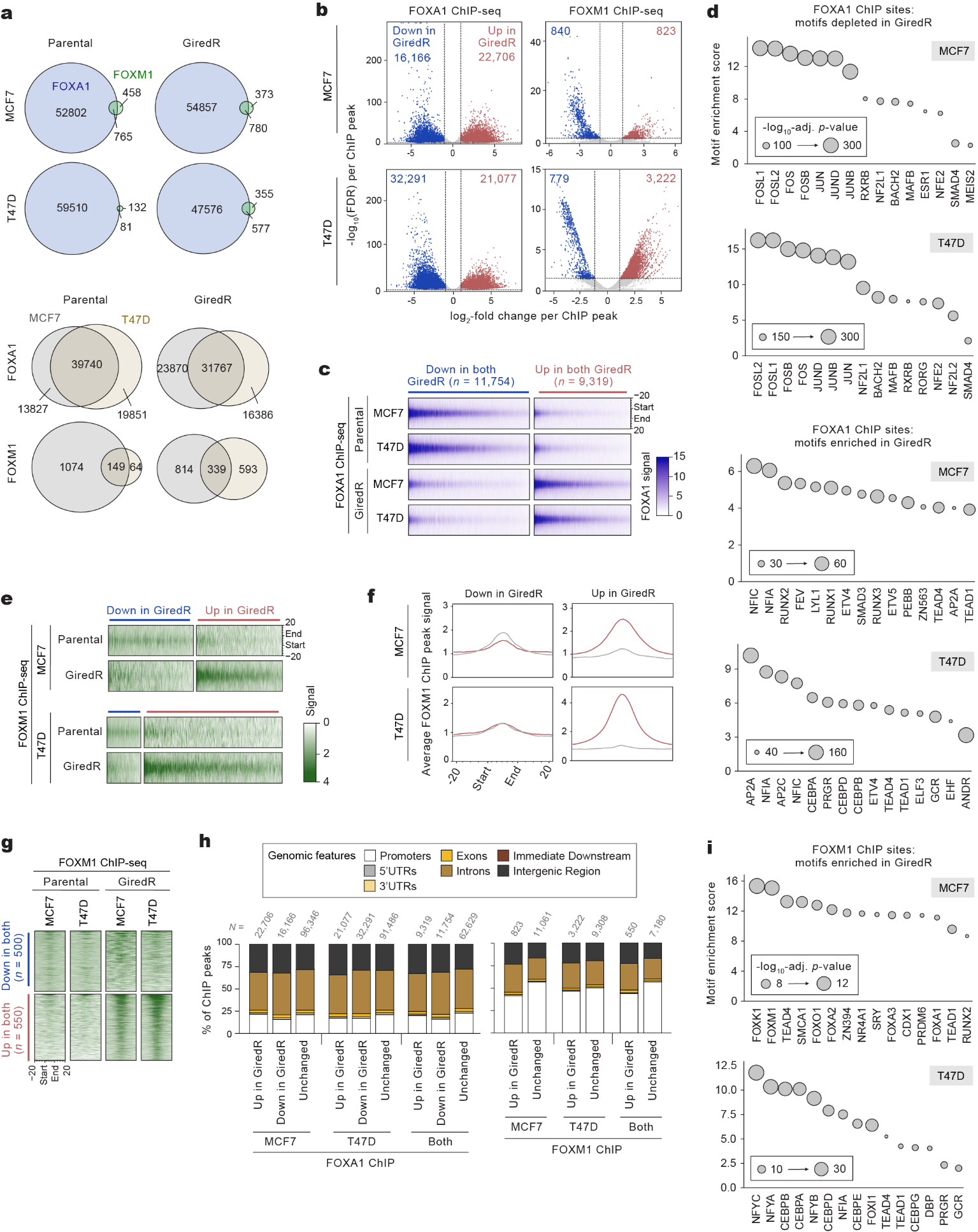


**Supplementary Figure 9. Additional FOXA1 and FOXM1 ChIP-seq analyses on GiredR cell lines.**

(**a**) Venn diagrams of high-confidence ChIP peaks for FOXA1 and FOXM1; *n =* 2 biological replicates per condition. High-confidence peaks were peaks identified in both biological replicates per condition. (Top) FOXA1 vs. FOXM1 per condition. (Bottom) MCF7 vs. T47D per TF and condition. (**b**) Volcano plots of differential FOXA1/FOXM1 ChIP-seq peaks in parental vs. GiredR cell lines. Each point is one ChIP peak. *N* = 135,218 and 144,854 FOXA1 peaks and *N* = 12,724 and 13,309 FOXM1 peaks for respective models. Dotted lines: log_2_-fold change of ± 1 and FDR < 0.05. (**c**) Heatmaps of differential FOXA1 ChIP-seq peaks common between both MCF7 and T47D in parental vs. GiredR cells. (**d**) For FOXA1 ChIP, motif enrichment analysis of GiredR-depleted motifs (down in GiredR) and GiredR-enriched motifs (up in GiredR). (**e-g**) Differential FOXM1 ChIP-seq sites in parental vs GiredR cells. (**e**) Heatmaps of differential ChIP-seq sites for FOXM1. (**f**) Average signal across ‘up in GiredR’ and ‘down in GiredR’ FOXM1 ChIP-seq peaks. (**g**) Heatmaps of differentially-accessible FOXM1 ChIP-seq peaks common between both MCF7 and T47D in parental vs. GiredR cells. (**h**) Genomic features for differential ChIP peaks per TF. Per TF and cell line, unchanged peaks were those which were present in both parental and GiredR without differential enrichment in either condition; *n* = 96,346 and 91,486 respectively for FOXA1 and *n* = 11,061 and 9,308 respectively for FOXM1; defined by -1 < log_2_-fold change < 1 and FDR < 1. (**i**) For FOXM1, motif enrichment analysis of GiredR-enriched motifs (up in GiredR) per cell line. In (**c, e-g**), one sample shown per condition, representative of *n* = 2 biological replicates. In (**h-i**), analyses corresponding to ‘down in GiredR’ FOXM1 peaks were omitted since these sites have low signal across conditions and ambiguous biological relevance; see (**e-f**). In (**d**, **i**), respective unchanged peaks were used as the reference for enrichment analysis. Each point is the enrichment score for one motif; *p*-values by one-sided Fisher’s exact test. Source data is available.

**
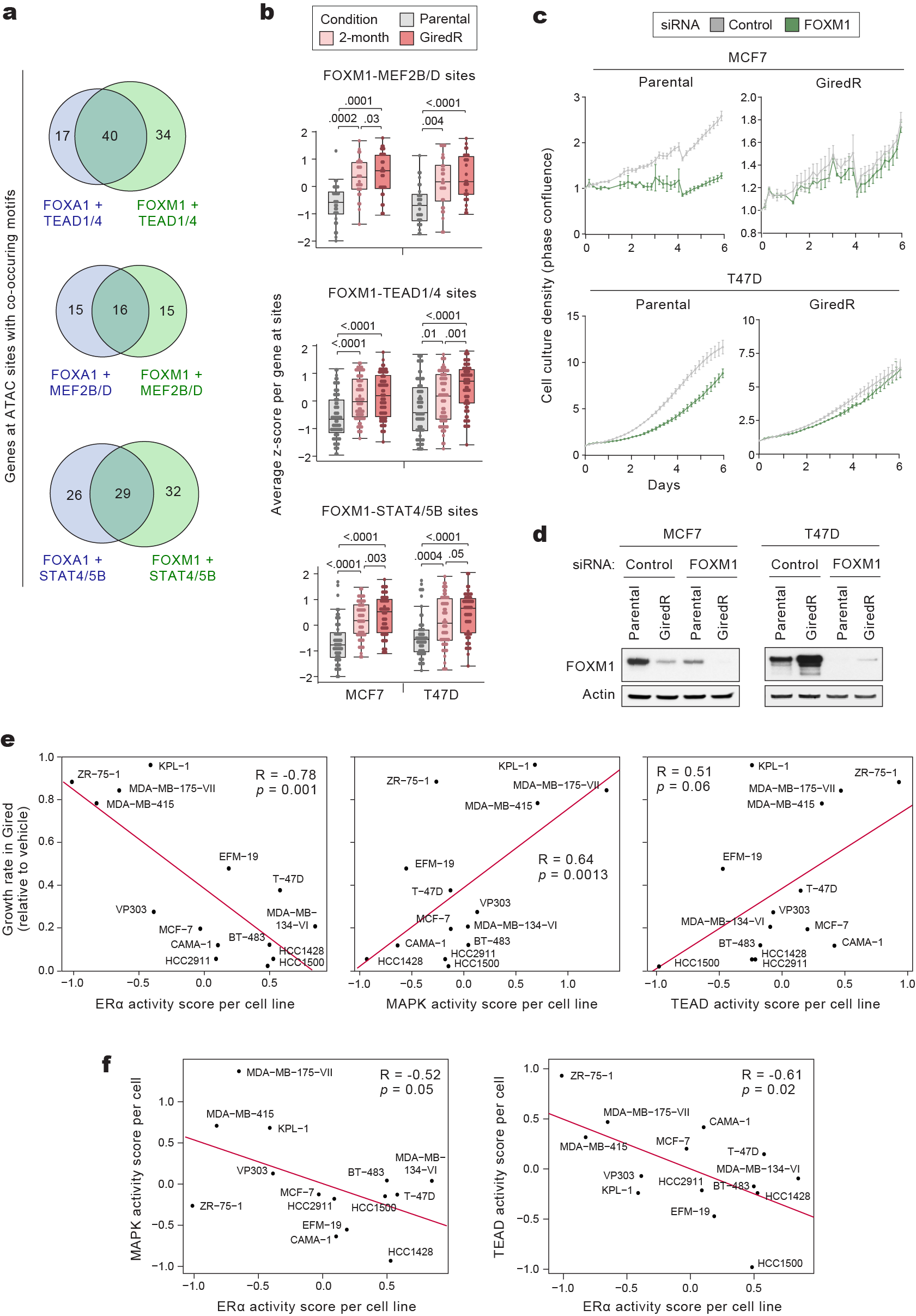
**

**Supplementary Figure 10. Additional analyses of FOXM1 and association between giredestrant resistance and orthogonal pathway activity.**

(**a**) For sites identified in Figure 4e with co-occurrence of FOXA1/FOXM1 and other motifs, the overlap of genes associated with FOXA1 versus FOXM1 co-occurrence. (**b**) For sites identified in Figure 4e with co-occurrence of FOXM1 and other motifs, the expression of genes at these sites per condition. Each point is one gene; genes were included in the analysis if the gene was proximal to >1 site with the respective motif pair. *N* = 29, 70, and 57 genes for FOXM1-MEF, TEAD, and STAT sites respectively. *P*-values by paired two-sided Mann-Whitney *U-*test, where each gene is paired across conditions. Boxes are 25^th^ percentile, median, and 75^th^ percentile, whiskers are maximum/minimum values within 1.5-times the IQR. (**c-d**) Effect of *FOXM1* knockdown on cell growth in parental and GiredR cells. (**c**) Cells were transfected with control or *FOXM1* siRNA, followed by growth rate measurements (Incucyte) for 6 days. Four *FOXM1* siRNAs were tested with similar results; data from one representative siRNA is shown. *N* = 3 biological replicates per condition; values are means ± S.D. (**d**) Western blot of cells collected after 6 days of Incucyte measurement to confirm *FOXM1* knockdown. (**e-f**) Association between growth rate in giredestrant and pathway activity for 14 ER+/HER2- breast cancer cell lines. Growth rate in giredestrant was computed using the GR method; *n* = 3 biological replicates were used to calculate growth rate per cell line. A value of 1.0 indicates that the given cell line grows equally well in both giredestrant and vehicle (DMSO) and a value of 0.0 indicates complete growth inhibition in the presence of giredestrant. Activity scores were calculated on *n* = 1 biological replicate per cell line; each point is one cell line. R and *p-*value via Pearson’s correlation method. (**e**) Growth rate in giredestrant versus pathway activity scores for ERα, MAPK, and TEAD for 14 ER^+^/HER2^-^ breast lines. (**f**) Comparison of ERα activity score versus other pathway activity scores for 14 ER^+^/HER2^-^ breast lines. Source data is available.

**Supplementary Table 1. List of local institutional review boards (IRBs) which reviewed and approved the study protocol for NCT03332797 (GO39932).**

| **Local institution’s IRB** | **Institution Location** |
| --- | --- |
| University of Colorado IRB | Aurora, Colorado, United States |
| Massachusetts General Hospital IRB | Boston, Massachusetts, United States |
| Beth Israel Deaconess Medical Center IRB | Boston, Massachusetts, United States |
| Dana Farber Cancer Institute IRB | Boston, Massachusetts, United States |
| Memorial Sloan Kettering Cancer Center IRB | New York, New York, United States |
| Vanderbilt University Medical Center IRB | Nashville, Tennessee, United States |
| St Vincent's Hospital Sydney IRB | Darlinghurst, New South Wales, Australia |
| Peter Maccallum Cancer Centre IRB | Melbourne, Victoria, Australia |
| National Cancer Center IRB | Gyeonggi-do, South Korea |
| Seoul National University Hospital IRB | Seoul, South Korea |
| Severance Hospital, Yonsei University IRB | Seoul, South Korea |
| Asan Medical Center IRB | Seoul, South Korea |
| Samsung Medical Center IRB | Seoul, South Korea |
| Hospital Quiron Barcelona IRB | Barcelona, Spain |
| Hospital Universitari Vall d'Hebron IRB | Barcelona, Spain |
| Hospital General Universitario Gregorio Maranon IRB | Madrid, Spain |
| Centro Oncologioco MD Anderson Internacional IRB | Madrid, Spain |
| Hospital Universitario Ramón y Cajal IRB | Madrid, Spain |
| Hospital Universitario HM Sanchinarro IRB | Madrid, Spain |
| Hospital Clinico Universitario de Valencia IRB | Valencia, Spain |
| ICO L'Hospitalet IRB | L'Hospitalet de Llobregat, Barcelona, Spain |
| Barts Health NHS Trust IRB | London, United Kingdom |
| The Royal Marsden Hospital IRB | Suttton, United Kingdom |
