# Supplementary material for: Loss of luminal lineage drives resistance to next-generation ERα antagonists in pretreated ER+ HER2− locally-advanced or metastatic breast cancer
Source: Nat Commun. 2026 Apr 1;17:4675. doi: 10.1038/s41467-026-71233-1 (PMC13201648; doi:10.1038/s41467-026-71233-1)
Supplement: Supplementary file 2 — Reporting Summary [file 41467_2026_71233_MOESM2_ESM.pdf]

nature portfolio

Corresponding author(s): Jackson Liang
Last updated by author(s): Mar 4, 2026

Reporting Summary

Nature Portfolio wishes to improve the reproducibility of the work that we publish. This form provides structure for consistency and transparency in reporting. For further information on Nature Portfolio policies, see our Editorial Policies and the Editorial Policy Checklist.

Statistics

For all statistical analyses, confirm that the following items are present in the figure legend, table legend, main text, or Methods section.

n/a	Confirmed	
☐	☒	The exact sample size (n) for each experimental group/condition, given as a discrete number and unit of measurement
☐	☒	A statement on whether measurements were taken from distinct samples or whether the same sample was measured repeatedly
☐	☒	The statistical test(s) used AND whether they are one- or two-sided<br>*Only common tests should be described solely by name; describe more complex techniques in the Methods section.*
☐	☒	A description of all covariates tested
☐	☒	A description of any assumptions or corrections, such as tests of normality and adjustment for multiple comparisons
☐	☒	A full description of the statistical parameters including central tendency (e.g. means) or other basic estimates (e.g. regression coefficient) AND variation (e.g. standard deviation) or associated estimates of uncertainty (e.g. confidence intervals)
☐	☒	For null hypothesis testing, the test statistic (e.g. F, t, r) with confidence intervals, effect sizes, degrees of freedom and P value noted<br>*Give P values as exact values whenever suitable.*
☒	☐	For Bayesian analysis, information on the choice of priors and Markov chain Monte Carlo settings
☒	☐	For hierarchical and complex designs, identification of the appropriate level for tests and full reporting of outcomes
☐	☒	Estimates of effect sizes (e.g. Cohen's d, Pearson's r), indicating how they were calculated

Our web collection on statistics for biologists contains articles on many of the points above.

Software and code

Policy information about availability of computer code

Data collection	No software was used for data collection.
Data analysis	For data analysis, the following software and platforms were used:<br>- Plotting and statistical analyses: Graphpad Prism 10<br>- Bioinformatics: R (v4.0 with RStudio)<br>- Publicly available packages: 'igis, ggplot2, DiffBind (v3), limma, ComplexHeatmap, profileplyr, chipenrich<br>- Custom packages: Genomics Platform packages ('gp.sa.core', 'gp.sa.diff', 'gp.sa.gsea'), 'FMItools'<br><br>Analysis code is available at https://doi.org/10.5281/zenodo.18644938. For ATAC-seq and ChIP-seq, all analysis code uses publicly-available R packages. For RNA-seq, some analyses code includes custom in-house functions. The custom code was designed to analyze RNA-seq data within our institution's cloud-based computing environment, and will not function properly outside of this environment. However, comparable analyses can be performed using publicly-available R packages.

For manuscripts utilizing custom algorithms or software that are central to the research but not yet described in published literature, software must be made available to editors and reviewers. We strongly encourage code deposition in a community repository (e.g. GitHub). See the Nature Portfolio guidelines for submitting code & software for further information.

Data

Policy information about availability of data

All manuscripts must include a data availability statement. This statement should provide the following information, where applicable:

- Accession codes, unique identifiers, or web links for publicly available datasets
- A description of any restrictions on data availability
- For clinical datasets or third party data, please ensure that the statement adheres to our policy

Raw and processed files from sequencing experiments have been uploaded to the NCBI Gene Expression Omnibus under the following accession IDs: GSE295127 (RNA-seq; www.ncbi.nlm.nih.gov/geo/query/acc.cgi?acc=GSE295127), GSE295128 (ATAC-seq; www.ncbi.nlm.nih.gov/geo/query/acc.cgi?acc=GSE295128), and GSE305432 (ChIP-seq; www.ncbi.nlm.nih.gov/geo/query/acc.cgi?acc=GSE305432). Due to data privacy laws and the terms of informed consent for study GO39932, raw data from patient-derived F-ACT and RNA-seq cannot be deposited in a public repository nor made available upon request. The remaining data for Figs. 1-4 and Supplementary figures have been provided as Source Data files.

Research involving human participants, their data, or biological material

Policy information about studies with human participants or human data. See also policy information about sex, gender (identity/presentation), and sexual orientation and race, ethnicity and racism.

Reporting on sex and gender	Sex or gender were not evaluated in this study, and are not reported.
Reporting on race, ethnicity, or other socially relevant groupings	Race, ethnicity, or other socially relevant groupings were not evaluated in this study, and are not reported.
Population characteristics	Age: Any (majority of patients were age 55 or older)<br>Gender: Female<br>Genotypic information: Patients were not genotyped as a criteria for enrollment for this clinical trial<br>Current diagnosis: locally-advanced or metastatic ER+/HER2- breast cancer<br>Treatment categories: giredestrant +/- palbociclib +/- LHRH agonist
Recruitment	Recruitment was performed by physicians at individual clinics, per physician's evaluation of eligibility. As described in Jhaveri, et al. (2023), key eligibility criteria were: "histologically/cytologically proven diagnosis of adenocarcinoma of the breast; evidence of either locally recurrent disease not amenable to resection or radiation therapy with curative intent or with metastatic disease; locally assessed ERpositivity (staining in ≥1% cells by immunohistochemistry) and HER2-negativity (per American Society of Clinical Oncology/College of American Pathologists) (30, 31); locally advanced/metastatic breast cancer that had recurred/progressed while being treated with adjuvant ET for ≥24 months and/or ET in the incurable/locally advanced/metastatic setting and derived a clinical benefit from ET (tumor response/stable disease for ≥6 months); ≤2 prior lines of treatment for locally advanced/metastatic breast cancer; ≥2 weeks elapsed from the use of any other ET/targeted therapy/chemotherapy; and postmenopausal women (pre-/perimenopausal women simultaneously received LHRH agonists with giredestrant 100mg)."<br><br>In addition, key exclusion criteria were: "known brain metastases that were untreated/symptomatic/required therapy to control symptoms; serious medical conditions/clinically significant abnormalities detected in clinical laboratory tests that precluded the patient's safe participation in and completion of the study; abnormal electrocardiogram, including complete left bundle branch block/second-/third-degree heart block/evidence of prior myocardial infarction; ongoing treatment with medications that prolong the QT interval; and, in a cardiac evaluation cohort (detailed below), ongoing treatment with medications that decrease heart rate (HR), including beta blockers."
Ethics oversight	As described in Jhaveri, et al. (2023), GO39932 "was conducted in accordance with the Declaration of Helsinki and Council for International Organizations of Medical Sciences International Ethical Guidelines, the International Council for Harmonisation Good Clinical Practice guidelines, or applicable laws and regulations of each country where the research was conducted if they provided greater protection to the individual. All patients provided written informed consent. The protocol, protocol amendments, informed consent form, Investigator Brochure, and other relevant documents (e.g., advertisements) were submitted to an Institutional Review Board (IRB)/ Independent Ethics Committee (IEC) by the investigator and reviewed and approved by the IRB/IEC before the study was initiated." This study was conducted in 23 clinical sites across the United States, Australia, South Korea, Spain and the United Kingdom; each clinical site reviewed the protocol via its own institution's ethics committee and criteria. See Supplementary Table 1 for full list of IRBs.

Note that full information on the approval of the study protocol must also be provided in the manuscript.

Field-specific reporting

Please select the one below that is the best fit for your research. If you are not sure, read the appropriate sections before making your selection.

☒ Life sciences ☐ Behavioural & social sciences ☐ Ecological, evolutionary & environmental sciences

For a reference copy of the document with all sections, see nature.com/documents/nr-reporting-summary-flat.pdf

Life sciences study design

All studies must disclose on these points even when the disclosure is negative.

Sample size	For cell line experiments, all assays (e.g. RPPA, RNAseq) were performed with 2-4 biological replicates as specified in respective figure captures. The sample size was determined based on prior experiments on the respective downstream measurements - these sample sizes have previously been shown to yield robust results on similar cell lines.
Data exclusions	No data were excluded.
Replication	Cell viability experiments were repeated 2-3 times per experiment (all successfully). For our sequencing experiments, which focused on profiling the baseline differences between established cell lines, experiments were performed once due to (1) budget limitations and (2) the fact that replication was unlikely to yield differences in baseline features for these cell lines.
Randomization	Randomization is not applicable to our cell line experiments, which focused on profiling two parental cell lines paired with their drug-resistant counterparts.
Blinding	No blinding was performed on the cell line experiments. Blinding was not applicable since all downstream analyses were comprised of either unbiased NGS profiling or instrument-based measurements on cell viability. Downstream computational approaches for sequencing data were also unbiased and applied equally to all samples evaluated.

Reporting for specific materials, systems and methods

We require information from authors about some types of materials, experimental systems and methods used in many studies. Here, indicate whether each material, system or method listed is relevant to your study. If you are not sure if a list item applies to your research, read the appropriate section before selecting a response.

Materials & experimental systems

n/a	Involved in the study
☐	☒ Antibodies
☐	☒ Eukaryotic cell lines
☒	☐ Palaeontology and archaeology
☒	☐ Animals and other organisms
☐	☒ Clinical data
☒	☐ Dual use research of concern
☒	☐ Plants

Methods

n/a	Involved in the study
☐	☒ ChIP-seq
☒	☐ Flow cytometry
☒	☐ MRI-based neuroimaging

Antibodies

Antibodies used	The following antibodies were used:<br>For Western blot: ERα (ThermoFisher, clone SP1, MA5-14501), FOXM1 (Cell Signaling, clone D3F2B, 20459), and β-actin (Cell Signaling, clone 8H10D10, 3700)<br>For ChIP-seq: anti-FOXA1 (Abcam, clone EPR10881, ab170933) and anti-FOXM1 (GeneTex, GTX102170)
Validation	All antibodies were validated by their respective manufacturers.<br>ER: https://www.thermofisher.com/antibody/product/Estrogen-Receptor-alpha-Antibody-clone-SP1-Monoclonal/MA5-14501<br>FOXM1: https://www.cellsignal.com/products/primary-antibodies/foxm1-d3f2b-rabbit-monoclonal-antibody/20459?srsltid=AfmBOoqHH4o7ryvzFQlp6mifirY85g86DtvzsvX-zWgaqQF6rB6g081Y<br>Actin: https://www.cellsignal.com/products/primary-antibodies/beta-actin-8h10d10-mouse-monoclonal-antibody/3700?srsltid=AfmBOop4wb19SLfsnMAGWEiK1wmT6fSKm-1YDIhj94kewlegz6y-nr2-<br>FOXA1 (ChIP-seq): https://www.abcam.com/en-us/products/primary-antibodies/foxa1-antibody-epr10881-ab170933<br>FOXM1 (ChIP-seq): https://www.genetex.com/Product/Detail/FOXM1-antibody/GTX102170?srsltid=AfmBOorWWY2poFDlk6lkTYIQu0AJHPLiNFgXi8Ns6Uo3j4lLjTVxRNBE

Eukaryotic cell lines

Policy information about cell lines and Sex and Gender in Research

Cell line source(s)	Both cell lines were sourced from ATCC: MCF7 (HTB-22) and T47D (HTB-133)
Authentication	Both cell lines were authenticated via STR profiling
Mycoplasma contamination	Both cell lines tested negative for Mycoplasma contamination
Commonly misidentified lines (See ICLAC register)	None

Clinical data

Policy information about clinical studies

All manuscripts should comply with the ICMJE guidelines for publication of clinical research and a completed CONSORT checklist must be included with all submissions.

Clinical trial registration	NCT03332797

Study protocol

This was a Phase Ia/Ib, multicenter, open-label, dose escalation and dose expansion study. The safety, pharmacokinetics, and activity of giredestrant (GDC-9545) was evaluated alone or in combination with palbociclib and/or LHRH agonists. Eligible patients had locally-advanced or metastatic ER+ breast cancer. Patients were enrolled onto the following study arms: (1) 10mg giredestrant, (2) 30mg giredestrant, (3) 90-100mg giredestrant, (4) 250mg giredestrant, and (5) 100mg giredestrant + 125mg palbociclib. Pre-menopausal patients were additionally administered LHRH agonists. All patients provided written informed consent to participate in this study. See also https://clinicaltrials.gov/ct2/show/NCT03332797.

Patient recruitment details:

Inclusion Criteria:

- Histologically or cytologically proven diagnosis of adenocarcinoma of the breast with evidence of either locally recurrent disease not amenable to resection or radiation therapy with curative intent or with metastatic disease
- Estrogen receptor (ER)-positive tumor
- Human epidermal growth factor receptor 2 (HER2)-negative breast cancer as per local laboratory testing
- Measurable disease, or evaluable bone disease; that is, bone lesions that are lytic or mixed (lytic + sclerotic) in the absence of measurable lesion
- Required paired pre- and on-treatment tumor biopsies for participants with metastases that are safely accessible as determined by the investigator
- Advanced or metastatic ER-positive/HER2-negative breast cancer that has recurred or progressed while being treated with adjuvant endocrine therapy for a duration of at least 24 months and/or endocrine therapy in the incurable, locally advanced, or metastatic setting and derived a clinical benefit from therapy (i.e., tumor response or stable disease for at least 6 months)
- No more than 2 prior lines of treatment for advanced or metastatic breast cancer
- Greater than or equal to (≥)2 weeks must have elapsed from the use of any other endocrine, targeted therapy or chemotherapy
- Single-Agent Cohorts (only applies to Dose Escalation): Advanced or metastatic disease that is either refractory to or intolerant of existing standard therapy or for which no effective standard therapy that confers clinical benefit is available
- Cohort B0: No prior treatment with cyclin-dependent kinase 4/6 (CDK4/6) inhibitor
- For participants undergoing 18F-fluoroestradiol-positron emission tomography (FES-PET) imaging additional restrictions on prior therapy include: ≥2 months must have elapsed from the use of tamoxifen; ≥6 months must have elapsed from the use of fulvestrant
- Postmenopausal status
- Eastern Cooperative Oncology Group (ECOG) Performance Status less than or equal to (≤)1
- Resolution of all acute toxic effects of prior therapy or surgical procedures to baseline or Grade ≤1 (except alopecia or other toxicities not considered to be a safety risk for the patient)
- Life expectancy of ≥12 weeks
- Adequate organ function

Exclusion Criteria:

- Known brain metastases that are untreated, symptomatic, or require therapy to control symptoms
- Current treatment with any systemic anti-cancer therapies for advanced disease (not applicable to Cohort X participants currently receiving GDC-0810 or GDC-0927)
- Concurrent treatment with warfarin or phenytoin
- Diagnosis of any secondary malignancy within 3 years prior to enrollment, except for appropriately treated carcinoma in situ of the cervix, non-melanoma skin carcinoma, or Stage I uterine cancer
- Active inflammatory bowel disease, chronic diarrhea, short bowel syndrome, or major upper gastrointestinal (GI) surgery including gastric resection
- Known human immunodeficiency virus (HIV) infection
- Known clinically significant history of liver disease consistent with Child-Pugh Class B or C, including active viral or other hepatitis (e.g., hepatitis B or hepatitis C virus), current alcohol abuse, or cirrhosis
- Major surgery within 4 weeks prior to enrollment
- Radiation therapy within 2 weeks prior to enrollment
- Other severe acute or chronic medical or psychiatric condition or laboratory abnormality that may increase the risk associated with study participation or investigational product administration or may interfere with the interpretation of study results and, in the judgment of the investigator, would make the patient inappropriate for entry into this study
- Inability or unwillingness to swallow tablets or capsules (only applies to Dose Escalation)
- Any serious medical condition or abnormality in clinical laboratory tests that, in the investigator's judgment, precludes the patient's safe participation in and completion of the study (only applies to Dose Escalation)
- History or presence of an abnormal electrocardiogram (ECG) that is clinically significant in the investigator's opinion, including complete left bundle branch block, second- or third-degree heart block, or evidence of prior myocardial infarction
- QT interval corrected using Fridericia's formula (QTcF) greater than (>)470 milliseconds (ms) demonstrated by at least two ECGs >30 minutes apart
- History of ventricular dysrhythmias or risk factors for ventricular dysrhythmias such as structural heart disease coronary heart disease clinically significant electrolyte abnormalities or family history of sudden unexplained death or long QT syndrome
- Current treatment with medications that are well known to prolong the QT interval

Data collection	Clinical follow-up was performed over the duration of the study to assess patient disease status and monitor possible adverse events. At the following visits - screening (baseline), C1D15 (cycle 1 day 15), C2D1, and disease progression - liquid biopsies were collected and tested by digital droplet PCR against key mutations and FoundationOne-Liquid CDx genomic profiling assays. Liquid biopsies were also used to measure giredestrant exposure. At screening and C2D1 visits, FFPE tissue blocks were collected to perform immunohistochemistry testing against ER, PR, and Ki67, and bulk RNA-sequencing.
Outcomes	Outcomes of this study were reported in the primary clinical publication: Khaveri, J, et. al. Phase Ia/b Study of Giredestrant ±Palbociclib and ±Luteinizing Hormone-releasing Hormone Agonists in Estrogen Receptor-positive, HER2-negative, Locally Advanced/ Metastatic Breast Cancer. (2023) Clin Cancer Res. (https://doi.org/10.1158/1078-0432.CCR-23-1796)<br><br>Key outcomes are summarized in the publication excerpt below:<br>"As of January 28, 2021, with 175 patients enrolled, no dose-limiting toxicity was observed, and the maximum tolerated dose was not reached. Adverse events (AEs) related to giredestrant occurred in 64.9% and 59.4% of patients in the single-agent ±LHRH agonist and giredestrant +palbociclib ±LHRH agonist cohorts, respectively (giredestrant-only related grade 3/4 AEs were reported in 4.5% of patients across the single-agent cohorts and 3.1% of those with giredestrant +palbociclib). Dose-dependent asymptomatic bradycardia was observed, but no clinically significant changes in cardiac-related outcomes: heart rate, blood pressure, or exercise duration. Clinical benefit was observed in all cohorts (48.6% of patients in the single-agent cohort and 81.3% in the giredestrant +palbociclib ±LHRH agonist cohort), with no clear dose relationship, including in patients with ESR1-mutated tumors."

Plants

Seed stocks	N/A
Novel plant genotypes	N/A
Authentication	N/A

ChIP-seq

Data deposition

- [x] Confirm that both raw and final processed data have been deposited in a public database such as GEO.
- [x] Confirm that you have deposited or provided access to graph files (e.g. BED files) for the called peaks.

Data access links<br>*May remain private before publication.*	https://www.ncbi.nlm.nih.gov/geo/query/acc.cgi?acc=GSE305432
Files in database submission	LIB6266388_SAM24489639_R1.fastq.gz<br>LIB6266389_SAM24489640_R1.fastq.gz<br>LIB6266392_SAM24489643_R1.fastq.gz<br>LIB6266393_SAM24489644_R1.fastq.gz<br>LIB6266396_SAM24489647_R1.fastq.gz<br>LIB6266397_SAM24489648_R1.fastq.gz<br>LIB6266400_SAM24489651_R1.fastq.gz<br>LIB6266401_SAM24489652_R1.fastq.gz<br>LIB6266404_SAM24489655_R1.fastq.gz<br>LIB6266405_SAM24489656_R1.fastq.gz<br>LIB6266408_SAM24489659_R1.fastq.gz<br>LIB6266409_SAM24489660_R1.fastq.gz<br>LIB6266412_SAM24489663_R1.fastq.gz<br>LIB6266413_SAM24489664_R1.fastq.gz<br>LIB6266416_SAM24489667_R1.fastq.gz<br>LIB6266417_SAM24489668_R1.fastq.gz<br>LIB6266420_SAM24489671_R1.fastq.gz<br>LIB6266422_SAM24489673_R1.fastq.gz<br>LIB6266424_SAM24489675_R1.fastq.gz<br>LIB6266426_SAM24489677_R1.fastq.gz<br>LIB6266388_SAM24489639_R2.fastq.gz<br>LIB6266389_SAM24489640_R2.fastq.gz<br>LIB6266392_SAM24489643_R2.fastq.gz<br>LIB6266393_SAM24489644_R2.fastq.gz<br>LIB6266396_SAM24489647_R2.fastq.gz

LIB6266397_SAM24489648_R2.fastq.gz
LIB6266400_SAM24489651_R2.fastq.gz
LIB6266401_SAM24489652_R2.fastq.gz
LIB6266404_SAM24489655_R2.fastq.gz
LIB6266405_SAM24489656_R2.fastq.gz
LIB6266408_SAM24489659_R2.fastq.gz
LIB6266409_SAM24489660_R2.fastq.gz
LIB6266412_SAM24489663_R2.fastq.gz
LIB6266413_SAM24489664_R2.fastq.gz
LIB6266416_SAM24489667_R2.fastq.gz
LIB6266417_SAM24489668_R2.fastq.gz
LIB6266420_SAM24489671_R2.fastq.gz
LIB6266422_SAM24489673_R2.fastq.gz
LIB6266424_SAM24489675_R2.fastq.gz
LIB6266426_SAM24489677_R2.fastq.gz
LIB6266388_SAM24489639_R1.srt.nodup_x_LIB6266420_SAM24489671_R1.srt.nodup.pval0.01.500K.bfilt.narrowPeak.gz
LIB6266389_SAM24489640_R1.srt.nodup_x_LIB6266420_SAM24489671_R1.srt.nodup.pval0.01.500K.bfilt.narrowPeak.gz
LIB6266392_SAM24489643_R1.srt.nodup_x_LIB6266422_SAM24489673_R1.srt.nodup.pval0.01.500K.bfilt.narrowPeak.gz
LIB6266393_SAM24489644_R1.srt.nodup_x_LIB6266422_SAM24489673_R1.srt.nodup.pval0.01.500K.bfilt.narrowPeak.gz
LIB6266396_SAM24489647_R1.srt.nodup_x_LIB6266424_SAM24489675_R1.srt.nodup.pval0.01.500K.bfilt.narrowPeak.gz
LIB6266397_SAM24489648_R1.srt.nodup_x_LIB6266424_SAM24489675_R1.srt.nodup.pval0.01.500K.bfilt.narrowPeak.gz
LIB6266400_SAM24489651_R1.srt.nodup_x_LIB6266426_SAM24489677_R1.srt.nodup.pval0.01.500K.bfilt.narrowPeak.gz
LIB6266401_SAM24489652_R1.srt.nodup_x_LIB6266426_SAM24489677_R1.srt.nodup.pval0.01.500K.bfilt.narrowPeak.gz
LIB6266404_SAM24489655_R1.srt.nodup_x_LIB6266420_SAM24489671_R1.srt.nodup.pval0.01.500K.bfilt.narrowPeak.gz
LIB6266405_SAM24489656_R1.srt.nodup_x_LIB6266420_SAM24489671_R1.srt.nodup.pval0.01.500K.bfilt.narrowPeak.gz
LIB6266408_SAM24489659_R1.srt.nodup_x_LIB6266422_SAM24489673_R1.srt.nodup.pval0.01.500K.bfilt.narrowPeak.gz
LIB6266409_SAM24489660_R1.srt.nodup_x_LIB6266422_SAM24489673_R1.srt.nodup.pval0.01.500K.bfilt.narrowPeak.gz
LIB6266412_SAM24489663_R1.srt.nodup_x_LIB6266424_SAM24489675_R1.srt.nodup.pval0.01.500K.bfilt.narrowPeak.gz
LIB6266413_SAM24489664_R1.srt.nodup_x_LIB6266424_SAM24489675_R1.srt.nodup.pval0.01.500K.bfilt.narrowPeak.gz
LIB6266416_SAM24489667_R1.srt.nodup_x_LIB6266426_SAM24489677_R1.srt.nodup.pval0.01.500K.bfilt.narrowPeak.gz
LIB6266417_SAM24489668_R1.srt.nodup_x_LIB6266426_SAM24489677_R1.srt.nodup.pval0.01.500K.bfilt.narrowPeak.gz
LIB6266388_SAM24489639_R1.srt.nodup_x_LIB6266420_SAM24489671_R1.srt.nodup.fc.signal.bigwig
LIB6266389_SAM24489640_R1.srt.nodup_x_LIB6266420_SAM24489671_R1.srt.nodup.fc.signal.bigwig
LIB6266392_SAM24489643_R1.srt.nodup_x_LIB6266422_SAM24489673_R1.srt.nodup.fc.signal.bigwig
LIB6266393_SAM24489644_R1.srt.nodup_x_LIB6266422_SAM24489673_R1.srt.nodup.fc.signal.bigwig
LIB6266396_SAM24489647_R1.srt.nodup_x_LIB6266424_SAM24489675_R1.srt.nodup.fc.signal.bigwig
LIB6266397_SAM24489648_R1.srt.nodup_x_LIB6266424_SAM24489675_R1.srt.nodup.fc.signal.bigwig
LIB6266400_SAM24489651_R1.srt.nodup_x_LIB6266426_SAM24489677_R1.srt.nodup.fc.signal.bigwig
LIB6266401_SAM24489652_R1.srt.nodup_x_LIB6266426_SAM24489677_R1.srt.nodup.fc.signal.bigwig
LIB6266404_SAM24489655_R1.srt.nodup_x_LIB6266420_SAM24489671_R1.srt.nodup.fc.signal.bigwig
LIB6266405_SAM24489656_R1.srt.nodup_x_LIB6266420_SAM24489671_R1.srt.nodup.fc.signal.bigwig
LIB6266408_SAM24489659_R1.srt.nodup_x_LIB6266422_SAM24489673_R1.srt.nodup.fc.signal.bigwig
LIB6266409_SAM24489660_R1.srt.nodup_x_LIB6266422_SAM24489673_R1.srt.nodup.fc.signal.bigwig
LIB6266412_SAM24489663_R1.srt.nodup_x_LIB6266424_SAM24489675_R1.srt.nodup.fc.signal.bigwig
LIB6266413_SAM24489664_R1.srt.nodup_x_LIB6266424_SAM24489675_R1.srt.nodup.fc.signal.bigwig
LIB6266416_SAM24489667_R1.srt.nodup_x_LIB6266426_SAM24489677_R1.srt.nodup.fc.signal.bigwig
LIB6266417_SAM24489668_R1.srt.nodup_x_LIB6266426_SAM24489677_R1.srt.nodup.fc.signal.bigwig

Genome browser session (e.g. UCSC)

No longer applicable

Methodology

Replicates

Two biological replicates per condition

Sequencing depth

For sequencing, a range of 30M to 70M reads were achieved for study samples. Across samples, the minimum number of unique reads were 20M and the average was ~30M unique reads. All samples have sequences of a single length (50bp). Reads were paired end.

Antibodies

Anti-FOXA1 (Abcam, EPR10881) and anti-FOXM1 (GeneTex, GTX102170)

Peak calling parameters

Peak calling was performed via the ENCODE ChIP-seq pipeline with MACS2 criteria. Per sample, the ChIP-seq FASTQ file was evaluated against a crosslinked chromatin input control.

Data quality

The following features were evaluated as measures of data quality: Normalized Strand Cross-correlation, Relative Strand Cross-correlation, and FRiP Score. For FOXA1 ChIP, 95% of peaks were at FDR 5% and 30% of peaks had 5-fold enrichment on average. For FOXM1 ChIP, 30% of peaks were at FDR 5% and 1% of peaks had 5-fold enrichment on average. (Note: Due to limitations in the FOXM1 ChIP data quality, the data were only presented in Supplementary Figures and were not used to draw any major conclusions for this study.)

Software | ChIP-seq data was analyzed in R via a combination of packages: DiffBind (v3), profileplyr, chipenrich
